# Incidence and determinants of low birth weight in Peninsular Malaysia: A multicentre prospective cohort study

**DOI:** 10.1371/journal.pone.0306387

**Published:** 2024-07-12

**Authors:** Elliza Mansor, Norliza Ahmad, Nor Afiah Mohd Zulkefli, Poh Ying Lim

**Affiliations:** 1 Ministry of Health, Putrajaya, Malaysia; 2 Department of Community Health, Faculty of Medicine and Health Sciences, Universiti Putra Malaysia, Serdang, Selangor, Malaysia; Karolinska Institutet, SWEDEN

## Abstract

**Background and objective:**

Pregnant mothers are at risk of many adverse pregnancy outcomes, including infants with low birth weight (LBW). The World Health Organization aimed to achieve a 30% reduction in the number of LBW infants by the year 2025. In this study, we aimed to determine the incidence and determinants of LBW infants among pregnant mothers attending government health clinics in Peninsular Malaysia.

**Material and methods:**

A prospective cohort study “Relative Risk of Determinants of Adverse Pregnancy Outcomes Among Pregnant Mothers Attending Government Health Clinics, Peninsular Malaysia, PEN-MUM” was conducted from March 2022 until March 2023 at 20 government health clinics in Peninsular Malaysia that were randomly selected through a multistage sampling method. Malaysian pregnant mothers between 18 and 49 years old were recruited at 12–18 weeks of gestation and followed up at three time points: 1 (24–28 weeks of gestation), 2 (36–40 weeks of gestation), and 3 (post-delivery). Eight exposure factors of LBW were studied: gestational weight gain, dengue infection, urinary tract infection, COVID-19 infection, gestational hypertension, preeclampsia, maternal anemia, and gestational diabetes mellitus (GDM).

**Results:**

Among 507 participants enrolled in the cohort, 40 were lost to follow-up. A total of 467 were included in the final analysis, giving an attrition rate of 7.9%. The incidence of LBW infants in Peninsular Malaysia was 14.3%. After adjusting for three covariates (ethnicity, employment status, and gestational age at birth), three determinants of LBW were identified. The risk of giving birth to LBW infants was higher among those with inadequate gestational weight gain (aRR = 2.86, 95% CI: 1.12, 7.37, p = 0.03), gestational hypertension (aRR = 4.12; 95% CI: 1.66, 10.43; p = 0.002), and GDM (aRR = 2.21; 95% CI: 1.18, 4.14; p = 0.013) during the second and third trimesters.

**Conclusions:**

The incidence of LBW infants in Peninsular Malaysia can be considered high. Having inadequate gestational weight gain, gestational hypertension, and GDM in the second and third trimesters increased the risk of LBW infants by threefold, fivefold, and twofold respectively. Thus, intervention strategies should target prevention, early detection, and treatment of gestational hypertension and GDM, as well as promoting adequate weight gain during antenatal care.

## Introduction

Low birth weight (LBW) refers to an infant weighing less than 2.5 kg at birth, regardless of gestational age [1]. Reducing the incidence of LBW birth is a top global commitment due to its high burden in many countries [2]. Globally, the prevalence of LBW was 14.7%, with some regions reporting a higher prevalence [3]. Southern Asia has the highest prevalence of LBW (26.4%), followed by sub-Saharan Africa (14%) and Southeast Asia (12%) [4]. In Malaysia, the national prevalence of LBW has plateaued over the years, with a prevalence of 10.9% in 2022 and 9.7% in 2016 [5]. However, it varied between states, ranging from 6.4% in 2016–2017 and 19.3% in 2020 reported in different studies [6]. The World Health Organization aimed to achieve a 30% reduction in the number of LBW infants by the year 2025. To achieve this target, the prevalence of LBW in Malaysia should be reduced to 8.0% and below [1].

The risk of early mortality is high among LBW infants. Apart from that, they are predisposed to other fetal complications such as neonatal respiratory distress syndrome and certain congenital abnormalities [7–9]. In Malaysia, the leading cause of neonatal mortality is “specific conditions originating in the perinatal period”, which include complications associated with LBW [10]. Hence, efforts to prevent LBW must be improved to reduce preventable deaths of children under five years old in this country. Children born as LBW infants are also more likely to experience issues with growth and development, such as stunting, wasting, as well as cognitive and motor delays [11–13]. Past studies have also established an increased risk of certain complications later in life, including metabolic disorders and chronic diseases such as coronary heart disease and stroke [14, 15]. For pregnant mothers, the birth of LBW infants can negatively impact their mental health, leading to anxiety and depression [16, 17]. These mothers have also been linked with other complications, such as venous thromboembolism and heart failure [18, 19]. Therefore, the birth of LBW infants affects the overall well-being of both children and mothers.

The pathophysiology of LBW is complex. So far, it has been shown that LBW infants suffer from reduced fetal growth before birth, possibly caused by a shortened gestation period, in-utero growth retardation, or both [20, 21]. Furthermore, maternal risk factors such as employment, marital status, preeclampsia, and specific health behaviors during pregnancy have also been shown to contribute to the development of LBW [22–25]. Hence, this study employed a prospective cohort study to identify the incidence and determinants of LBW among Malaysian pregnant mothers. Consistent with the current non-communicable diseases’ health burden in Malaysia, an increased prevalence of hypertension and diabetes among pregnant mothers has also been reported in the past few years [5]. Thus, this study also aimed to determine common medical conditions and obstetric-related problems that can predispose to LBW [26, 27].

## Materials and methods

### Study design, population, and sample size

A prospective cohort study, “Relative Risk of Determinants of Adverse Pregnancy Outcomes Among Pregnant Mothers Attending Government Health Clinics, Peninsular Malaysia,” or PEN-MUM was conducted in 20 government health clinics in Peninsular Malaysia. Peninsular Malaysia comprises 11 states and 2 federal territories [28, 29].

Eligibility criteria included Malaysian mothers registered with the government health clinic, aged 18 to 49, between 12 and 18 gestational weeks, and having a smartphone with an internet connection. Those with multiple pregnancies, miscarriages, diagnosed with intrauterine growth restriction, unconfirmed estimated delivery date (EDD), chronic medical disorders, or discontinued antenatal visits were excluded. The sample size needed was 630 participants after considering a 95% confidence level, 80% power, and 30% non-response rate. It was calculated using the sample size calculation formula for longitudinal studies [30] with two proportions based on a prior study [31].

### Sampling method

A multistage random sampling was applied to select 20 health clinics in Peninsular Malaysia. Then, the probability proportional to size (PPS) sampling was applied to each clinic based on the sample size calculated. Finally, a systematic random sampling was used to select eligible participants from each health clinic.

### Study instruments

This study utilized two study instruments: a digital self-administered questionnaire and a physical standard data collection form. The digital self-administered questionnaire captured data on maternal socio-demographic characteristics, i.e. age, ethnicity, education level, employment status, marital status, monthly household income, residency area, and the type of cultural practice. Meanwhile, a physical data collection form was used to collect maternal health information on LBW outcomes, obstetric-related problems (gestational weight gain, parity, gestational age at booking, maternal height, maternal body mass index [BMI], and gestational age at birth), and specific medical conditions (dengue infection, urinary tract infection, COVID-19 infection, gestational hypertension, preeclampsia, maternal anemia, and GDM).

Gestational weight gain is the recommended weight gain as classified by the Institute of Medicine (IOM). In this study, it was computed from pregnant mothers’ weight recorded in the maternal health record at each time point and further categorized as inadequate, adequate, and excessive weight gain based on maternal BMI: underweight (12.5–18.0 kg), normal (11.5–16.0 kg), overweight (7.0–11.5 kg), and obese (5.0–9.0 kg) [32]. Meanwhile, the coexistence of medical conditions was marked as ‘yes/no’ based on the presence of the problems at each time point as per the maternal health book record. The quality control for the study instruments was ensured by performing content validity and face validity. A one-week test-retest reliability showed good agreement (Cohen’s Kappa: 0.72–0.86).

### Study variables

#### Outcome variable

The outcome variable was LBW, defined as a birth weight of less than 2500 g [1].

#### Exposure variables

The exposure variables in this study included gestational weight gain, dengue infection, urinary tract infection, COVID-19 infection, gestational hypertension, preeclampsia, maternal anemia, and GDM. The operation definition for each exposure variable is listed in S1 Appendix.

### Data collection

The recruitment period started from March 1, 2022 until July 31, 2022. Data collection for the cohort continued for one year until March 2023. All eligible pregnant mothers between the gestation age of 12 and 18 weeks were approached. Those who provided written informed consent were given the link to the digital questionnaire to be answered on their digital devices. As the data collection was conducted during the COVID-19 outbreaks, the digital questionnaire was used to minimize direct contact between participants and healthcare personnel.

In addition, the participants’ maternal health records were traced and captured using a standard data collection form by the study investigators. The cohort was followed up at three specific time points, i.e., Point 1 (24–28 weeks of gestation), Point 2 (36–40 weeks of gestation) for the changes in study exposure, and Point 3 (post-delivery) for the study outcome. The researchers identified individual participants through their identification numbers to match the data from the maternal health record and the digital questionnaires. All data were recorded on a password-protected laptop and could only be assessed by the researchers.

### Statistical analysis

The data were analysed using IBM SPSS version 26.0. Prior to analysis, data sets were examined for missing data, outliers, and normality. Continuous data were presented as mean and standard deviations (normal distribution) or median and interquartile range (non-normal distribution) before being further assessed using an independent t-test. All categorical data were presented in frequency and percentage and compared by chi-square or Fisher’s exact tests. For bivariate analysis, simple logistic regression was employed. Finally, a generalized linear mixed model with a binary logit function was used to identify the determinants of LBW. The final model was adjusted with covariates, i.e., ethnicity, employment status, and gestational age at birth. Health clinics were selected as a random effect and the best model was chosen using a forward stepwise selection based on a smaller Akaike Information Criterion (AIC) value. All results were presented as relative risk (RR) with 95% confidence intervals (CI) and the level of significance was set at P <0.05.

### Ethics statement

Ethical approval was granted by the National Medical Research and Ethics Committee (NMREC) of the National Institute of Health, Ministry of Health Malaysia (NMRR-21-1789-61052) dated 27 October 2021. Permission was also obtained from the selected State Health Departments (Selangor, Negeri Sembilan, Pahang, and Penang). Upon recruitment, written informed consent was acquired from the participants. They were also informed that all information would be kept confidential and that they could withdraw from the study at any time.

## Results

### Sample characteristics

**Fig 1:**
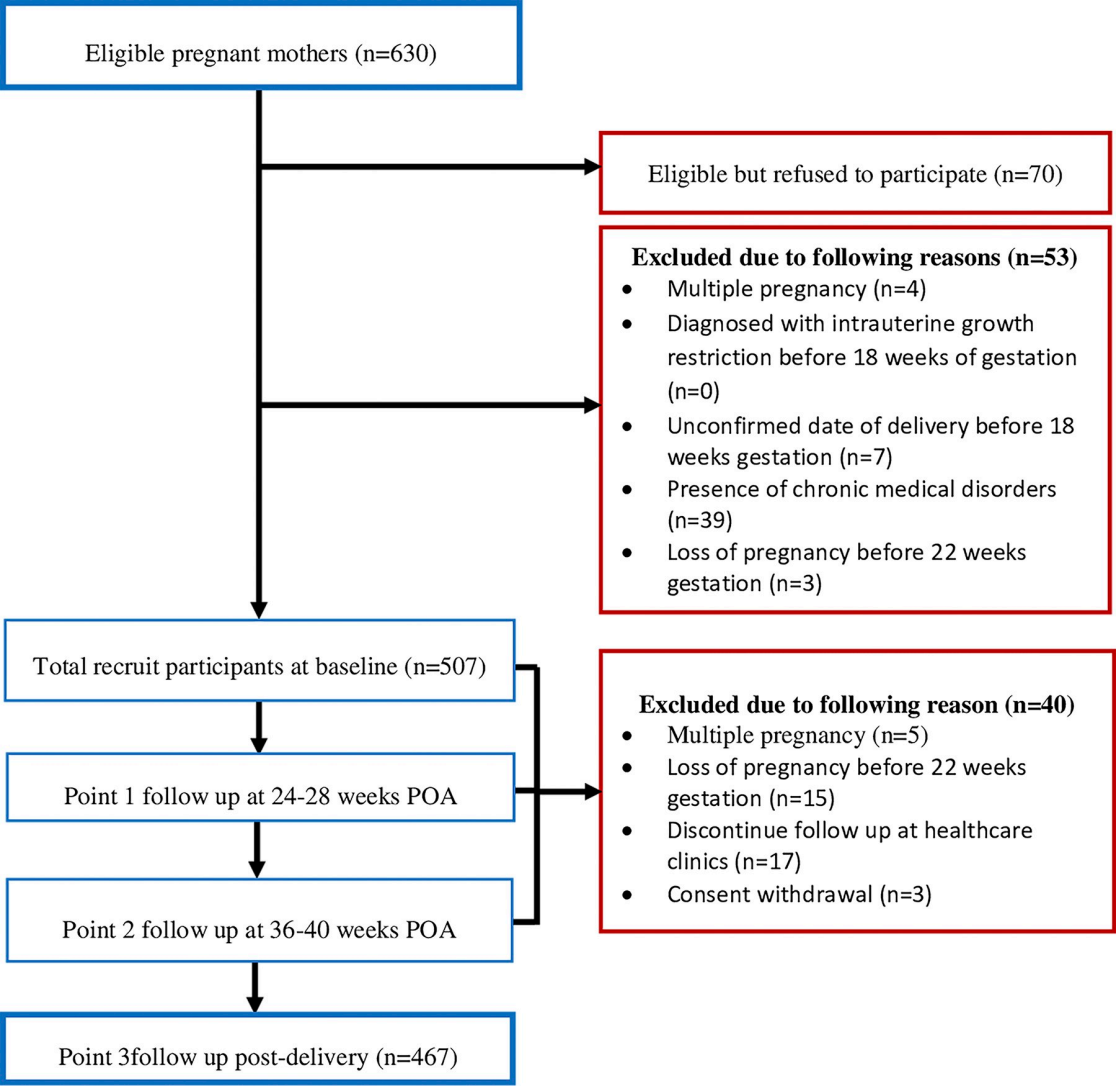
Study flow and recruitment.

At the baseline, a total of 507 participants were recruited from 630 pregnant mothers who were approached, giving a response rate of 80.5%. However, over the one-year follow-up, 40 were excluded due to: multiple pregnancies (n = 5), miscarriage (n = 15), discontinued follow-up at government health clinics (n = 17), and consent withdrawal (n = 3). Thus, only 467 participants were included in the final analysis, giving an attrition rate of 7.9% (Fig 1).

Table 1 shows the characteristics of the study participants. The mean (SD) age of pregnant mothers was 29.6 (4.90) years, with the majority being Malays (88.0%). Moreover, ethnicity was significantly associated with the risk of giving birth to LBW infants (χ2 = 4.07, df = 1, p = 0.044). Meanwhile, more than half of the study participants had high education levels (54.8%) of either a diploma, certificate or higher. Employment status was also associated with LBW among pregnant mothers in this cohort (χ2 = 9.65, df = 2, p = 0.008). Approximately 98.7% were married. The median (IQR) monthly household income was RM 3,000 (2,000). Most of the participants (81.4%) stayed in urban residency areas. Interestingly, a big proportion of pregnant mothers (70.0%) in this cohort practiced food taboos or used only traditional medicine. Meanwhile, most mothers were multiparous (55.2%) and came to healthcare clinics for early booking (71.7%). The median (IQR) of the participants for maternal height was 156.0 cm (8.0) and nearly half of them had a normal BMI (46.5%). For gestational age at birth, most delivered a term infant (94.2%). This factor was also associated with an increased risk of giving birth to LBW infants (p < 0.001).

**Table 1:** Socio-demographic and maternal characteristics of pregnant mothers and their association with LBW.

Covariates	LBW (n = 67)	Non-LBW (n = 400)	All (n = 467)	t-statistic (df) / χ2 (df)	P value
	n (%) Mean ± SD/ Median (IQR)	n (%) Mean ± SD/ Median (IQR)	n (%) Mean ± SD/ Median (IQR)		
**Age**	29.79 ± 4.69	29.62 ± 4.94	29.64 ± 4.90	-0.26 (465)	0.792
**Ethnicity**				4.07 (1)	0.044
Non-Malay^a^	13 (23.2)	43 (76.8)	56 (12.0)		
Malay	54 (13.1)	357 (86.9)	411 (88.0)		
**Education level**				0.01 (1)	0.942
Low education^b^	30 (14.2)	181 (85.8)	211 (45.2)		
High education^c^	37 (14.5)	219 (85.5)	256 (54.8)		
**Employment status**				9.65 (2)	0.008
Unemployed	18 (10.0)	162 (90.0)	180 (38.6)		
Self-employed	1 (3.3)	29 (96.7)	30 (6.4)		
Employed	48 (18.7)	209 (81.3)	257 (55.0)		
**Marital status**					
Unmarried/Widowed/Separated	1 (16.7)	5 (83.3)	6 (1.3)	–^e^	1.00
Married	66 (14.3)	395 (85.7)	461 (98.7)		
**Monthly household income (RM)**	2500 (2000)	3000 (2000)	3000 (2000)	0.67 (465)	0.501
**Residency area**				1.39 (1)	0.238
Rural	9 (10.3)	78 (89.7)	87 (18.6)		
Urban	58 (15.3)	322 (84.7)	380 (81.4)		
**Type of cultural practices** ^**d**^				2.17 (2)	0.339
Practicing both	4 (11.4)	31 (88.6)	35 (7.5)		
Practicing either one	52 (15.9)	275 (84.1)	327 (70.0)		
Not practicing both	11 (10.5)	94 (89.5)	105 (22.5)		
**Parity**				1.14 (1)	0.286
Nulliparity	34 (16.3)	175 (83.7)	209 (44.8)		
Multiparity	33 (12.8)	225 (87.2)	258 (55.2)		
**Gestational age at booking**				2.10 (1)	0.148
Late booking	14 (10.6)	118 (89.4)	132 (28.3)		
Early booking	53 (15.8)	282 (84.2)	335 (71.7)		
**Maternal height (cm)**	154.0 (5.0)	156.0 (8.0)	156.0 (8.0)	1.22 (465)	0.223
**Maternal BMI**	23.94 (6.89)	24.24 (8.25)	24.1 (8.0)	1.84 (3)	0.607
Underweight	6 (13.6)	38 (86.4)	44 (9.4)		
Normal	36 (16.6)	181 (83.4)	217 (46.5)		
Overweight	13 (11.4)	101 (88.6)	114 (24.4)		
Obese	12 (13.0)	80 (87.0)	92 (19.7)		
**Gestational age at birth**				–^e^	<0.001
Preterm	16 (59.3)	11 (40.7)	27 (5.8)		
Term	51 (11.6)	389 (88.4)	440 (94.2)		

Data are presented n (%), mean ± SD, median (IQR) and RR. Bold denotes statistical significance.

t-statistic: independent t-test, χ2: chi-square, df: degree of freedom, ref: reference, RM: Ringgit Malaysia, cm: centimeter.

^a^ Non-Malay includes Chinese, Indian and other ethnicity in Malaysia.

^b^ Low education includes those with no formal education, primary and secondary education.

^c^ High education includes those with diploma/certificate, bachelor’s degree and master’s/PhD

^d^ Cultural practice includes food taboos and traditional medications used

^e^ Fisher exact test

The exposure status of the pregnant mothers is summarized in Table 2. Based on the IOM classification of recommended weight gain during pregnancy, most participants recorded inadequate weight gain at all time points (43.3% observed in total). Pregnant mothers infected with dengue, urinary tract infection, and COVID-19 during pregnancy were 0.4%, 11.6%, and 16.3% respectively. Most of them were diagnosed with gestational hypertension after 28 weeks of gestation (5.8%). Likewise, 1.7% of all cases of preeclampsia were diagnosed during that period. The mean (SD) of hemoglobin level was 10.8 ± 0.95 g/dL, with almost half of the participants having maternal anemia at any point in pregnancy (52.7%). The proportion of GDM was also high, with more than half (52.7%) of the participants having GDM.

**Table 2:** Exposure status of pregnant mothers.

Exposure variables	Baseline (12–18 weeks) (n = 467)	Point 1 (24–28 weeks) (n = 466)	Point 2 (36 weeks-delivery) (n = 453)	All (n = 467)
	n (%) mean ± SD	n (%) mean ± SD	n (%) mean ± SD	n (%) mean ± SD
**Gestational weight gain (kg)**	1.64 ± 3.14	4.47 ± 2.55	4.33 ± 2.68	10.44 ± 5.64
Inadequate	269 (57.6)	72 (15.5)	118 (26.1)	202 (43.3)
Adequate	68 (14.6)	61 (13.1)	98 (21.6)	156 (33.4)
Excessive	130 (27.8)	333 (71.4)	237 (52.3)	109 (23.3)
**Dengue infection**				
Yes	0 (0)	1 (0.2)	1 (0.2)	2 (0.4)
No	467 (100)	465 (99.8)	452 (99.8)	465 (99.6)
**Urinary tract infection**				
Yes	27 (5.8)	18 (3.9)	9 (2.0)	54 (11.6)
No	440 (94.2)	448 (96.1)	444 (98.0)	413 (88.4)
**COVID-19 infection**				
Yes	37 (7.9)	13 (2.8)	26 (5.7)	76 (16.3)
No	430 (92.1)	453 (97.2)	427 (94.3)	391 (83.7)
**Gestational hypertension**				
Yes	0 (0)	4 (0.9)	25 (5.5)	27 (5.8)
No	467 (100)	462 (99.1)	428 (94.5)	440 (94.2)
**Preeclampsia**				
Yes	0 (0)	0 (0)	8 (1.8)	8 (1.7)
No	467 (100)	466 (100)	445 (98.2)	459 (98.3)
**Maternal anemia (Hb in g/dL)**	11.61 ± 1.11	11.22 ± 0.96	11.65 ± 0.95	10.8 ± 0.95
Yes	112 (24.0)	168 (36.0)	97 (21.4)	246 (52.7)
No	355 (76.0)	298 (64.0)	356 (78.6)	221 (47.3)
**Gestational diabetes mellitus**				
Yes	189 (40.5)	246 (52.8)	233 (51.4)	246 (52.7)
No	278 (59.5)	220 (47.2)	220 (48.6)	221 (47.3)

Data are presented n (%) and mean ± SD.

kg: kilogram. Hb: hemoglobin, g/dL: grams per deciliter.

### Incidence of LBW infants and its determinants

The overall incidence of LBW among pregnant mothers attending government health clinics in Peninsular Malaysia was 14.3% (n = 67). The Generalized Linear Mixed (GLM) Model was used to analyze the determinants of LBW infants, with level 1 representing pregnant mothers (n = 467) and level 2 representing government health clinics (n = 20). Four exposure variables with p < 0.25 from the bivariate analysis were included in the multivariate analysis: gestational weight gain, dengue infection, gestational hypertension, and GDM. Three determinants were identified from the multivariate analysis (Table 3). Pregnant mothers with inadequate weight gain during pregnancy recorded a three times higher risk of giving birth to LBW infants (adjusted RR = 2.86, 95% CI: 1.12,7.37, p = 0.03). Two medical conditions were also significantly associated with the risk of LBW infants. Mothers with gestational hypertension diagnosed at the second or third trimesters in pregnancy were five times at risk of delivering LBW infants than their counterparts (adjusted RR = 4.66, 95% CI: 1.88,11.55, p = 0.001). Meanwhile, those with GDM were also twice as likely to give birth to infants with LBW than those without GDM (adjusted RR = 2.27, 95% CI: 1.26,4.08, p = 0.006).

**Table 3:** Association between exposure variables and LBW infants (N = 467).

Exposure Variables	LBW (n = 67)	Non-LBW (n = 400)	Bivariate analysis		Multivariate analysis	
	n (%) Mean ± SD	n (%) Mean ± SD	RR (95% CI)	P value	aRR (95% CI)	P value
**Intercept**					0.52	
**Gestational weight gain (kg)**	8.05 ± 5.12	10.58 ± 5.74				
Inadequate	40 (19.8)	162 (80.2)	1.68 (0.94, 3.01)	0.082	2.86 (1.12,7.37)	0.030
Excessive	7 (6.4)	102 (93.6)	0.47 (0.19, 1.15)	0.096	0.72 (0.38,1.35)	0.303
Adequate	20 (12.8)	136 (87.2)	Ref		Ref	
**Intercept**					-0.63	
**Dengue infection**						
Yes	1 (50.0)	1 (50.0)	6.05 (0.37,97.84)	0.205	1.32 (0.05,32.28)	0.863
No	66 (14.2)	399 (99.8)	Ref		Ref	
**Urinary tract infection**						
Yes	5 (9.3)	49 (90.7)	0.58 (0.22, 1.51)	0.262	NT	
No	62 (15.0)	351 (85.0)	Ref			
**COVID-19 infection**						
Yes	11 (14.5)	65 (85.5)	1.01 (0.50,2.04)	0.973	NT	
No	56 (14.3)	335 (83.6)	Ref			
**Intercept**					-1.85	
**Gestational hypertension**						
Yes	10 (37.0)	17 (63.0)	3.95 (1.73, 9.06)	0.001*	4.66 (1.88,11.55)	0.001
No	57 (13.0)	383 (87.0)	Ref		Ref	
**Preeclampsia**						
Yes	1 (12.5)	7 (87.5)	0.85 (0.10,7.03)	0.881	NT	
No	66 (14.4)	393 (85.6)	Ref			
**Maternal anemia (Hb in g/dL)**	10.95 ± 1.13	10.83 ± 0.91				
Yes	33 (13.4)	213 (86.6)	0.85 (0.51,1.43)	0.545	NT	
No	34 (15.4)	187 (84.6)	Ref			
**Intercept**					-0.83	
**Gestational diabetes mellitus**						
Yes	46 (18.7)	200 (81.3)	2.19 (1.26, 3.81)	0.005*	2.27 (1.26,4.08)	0.006
No	21 (9.5)	200 (90.5)	Ref		Ref	

Data are presented as n (%), mean ± SD, RR and aRR. Bold P value denotes statistical significance.

Bivariate analysis: simple logistic regression, multivariate analysis: generalized linear mixed model (Akaike corrected value: 2385.738–2441.938 and Bayesian corrected value: 2389.863–2446.058).

kg: kilogram. Hb: hemoglobin, g/dL: grams per deciliter, RR: relative risk, aRR: adjusted relative risk, ref: reference, NT: not tested variables, those not included in multivariable analysis.

## Discussion

LBW remains a major health concern globally due to its impact on the well-being of infants and mothers. This study observed specific exposure factors present in the second and third trimesters of pregnancy toward the risk of giving birth to LBW infants among Malaysian mothers. Compared to previous studies [7, 8], this study was conducted in the more populated areas of the country. As this study is a cohort study, we reported the incidence instead of the prevalence. The incidence of LBW infants obtained from this study was 14.3%, consistent with the global prevalence of 14.6% reported in 2015 (3). However, the incidence was higher than the overall prevalence in the Southeast Asia region, i.e., 12.2% [4]. This finding might indicate an increasing trend of LBW infants being born in Malaysia in recent years. Based on the National Health and Morbidity Survey (NHMS) 2022, the overall prevalence of LBW in 2022 was 10.9% compared to 9.7% in 2016 [5, 6]. Furthermore, to achieve the WHO target, the prevalence of LBW in 2025 should be reduced to 8.0% and below, i.e., a 30% reduction compared with the prevalence of LBW infants in Malaysia in 2012 [2]. Based on our findings, more effort should be effectively implemented to reduce adverse outcomes associated with LBW. Nevertheless, the incidence measured in this study may not reflect the national incidence as it was only conducted in Peninsular Malaysia. Furthermore, compared with the NHMS 2022 that was conducted among children under five years old, our study observed the LBW outcomes of mothers who were recruited during the early second trimester.

With regard to the determinants of LBW, inadequate gestational weight gain based on the 2009 IOM classification was one of the determinants of LBW identified among local mothers in this study. This observation is consistent with previous studies [26, 33, 34]. Gestational weight gain is often related to maternal nutritional status [35]. Several local studies have documented a significant prevalence of inadequate weight gain among pregnant mothers, one of the common obstetric problems in pregnancy [36, 37]. During pregnancy, the growing fetus depends on the mother for essential nutrients, such as proteins, carbohydrates, fats, vitamins, and minerals, for its development. Therefore, inadequate weight gain may result in an inadequate intake of these nutrients, leading to negative effects on fetal growth in utero and the birth of LBW infants [35, 38]. Poor weight gain during pregnancy can also lead to placental insufficiency, subsequently compromising the placenta’s efficiency in transporting nutrients from the mother to the fetus and directly impacting normal fetal growth [39, 40].

In addition, the risk of having LBW infants was found to be almost five-fold greater among those with gestational hypertension in this study, consistent with those reported by a meta-analysis [41]. This relationship is primarily attributable to the complex biochemical alterations that happen in pregnant mothers with hypertension. Various genetic and environmental factors can uteroplacental maladaptation, leading to placentation defects, decreased uteroplacental perfusion pressure, and placental ischemia [42]. Placental ischemia causes the release of specific bioactive factors, such as inflammatory cytokines, reactive oxygen species, and hypoxia-inducible factors in mothers with hypertensive disorders in pregnancy. Collectively, these factors may cause a decrease in uteroplacental blood flow and subsequently lead to fetal growth restriction [42, 43]. Such growth restriction could further complicate the outcomes of LBW infants [20, 44].

As for GDM, past research indicates that GDM significantly affects infant birth weight but mainly towards macrosomia (high birth weight) [45]. A meta-analysis reported that no clear evidence was found to support the delivery of LBW infants by GDM mothers. This finding was inconsistent with our study [45]. A plausible explanation for our results could be maternal microangiopathy or diabetes-related damage to small blood vessels. Microangiopathy can reduce uteroplacental blood flow, resulting in fetal hypoxia and inadequate nutrition, which further activate a series of metabolic and hormonal alterations in the fetus, ultimately resulting in abnormal fetal growth and LBW [46, 47]. Another reason could be related to lifestyle adjustments, including diet and physical activity, which may also reduce fetal growth and weight in utero [48]. Mothers with GDM may also engage in excessive dietary restrictions or physical activity out of fear of diabetes-related complications, thus negatively impacting fetal growth and resulting in LBW [49, 50].

The strengths of our study included the prospective cohort study design that involved 20 clinics in Peninsular Malaysia using multistage random sampling, thus improving the generalizability of our study findings. Furthermore, the follow-up nature of the cohort study limited the measurement bias between the exposure variables and the outcome. In addition, the determinants of LBW infants were measured using the GLM model that considered the random and fixed variables, thus more robust.

However, our study has several limitations. Firstly, the eligibility criteria of possessing a smartphone with an internet connection and the exclusion criteria of multiple pregnancies could have resulted in a selection bias. Pregnant mothers without smartphones, either due to low socio-economic status or being technology illiterate were excluded. In addition, data collected using digital questionnaires could have resulted in some difficulty for the participants, especially if there were connectivity issues. Besides, this study did not capture previous obstetric history of delivering infants with LBW and we did not assess outcomes of the babies born in this cohort especially those with low birth weight. There was a bias in identifying disease conditions that could lead to LBW without considering constitutional factors. Lastly, the maternal nutrition was not captured and evaluated throughout the three time points which could contributed to the LBW.

## Conclusion

In this study, the incidence of LBW in Peninsular Malaysia was 14.3%, higher than the local target of 8% aspired to be achieved by 2025. Therefore, further efforts are needed to reduce this public health burden in Malaysia. The risk of having LBW infants was found to increase by three-fold for mothers with inadequate gestational weight gain, as well as five-fold and two-fold for mothers diagnosed with gestational hypertension and GDM in the second or third trimesters of pregnancy. In view of this, targeted intervention efforts to promote adequate weight gain, prevention, early detection, and treatment of gestational hypertension and GDM should be initiated early as a part of pre-pregnancy or early pregnancy management.

## Supporting information

S1 Appendix Operational definition. (DOCX)
