# Supplementary material for: Incidence and determinants of low birth weight in Peninsular Malaysia: A multicentre prospective cohort study
Source: PLoS One. 2024 Jul 12;19(7):e0306387. doi: 10.1371/journal.pone.0306387 (PMC11244833; doi:10.1371/journal.pone.0306387)
Supplement: S1 Appendix — (DOCX) [file pone.0306387.s001.docx]

**Operational definition for exposure variables**

| Variables | Operational Definition |
| --- | --- |
| Gestational weight gain | It refers to recommended weight gain by IOM classification computed for pregnant mothers based on maternal BMI, categorised as below:  Rate of gestational weight gain in the second and third trimesters was defined as the average weekly weight gain in that trimester (mean (range) in kg/week):   1. BMI underweight: 0.51 (0.44-0.58) 2. BMI normal: 0.42 (0.35-0.50) 3. BMI overweight: 0.28 (0.23-0.33) 4. BMI obese: 0.22 (0.17-0.27)   Total gestational weight gain was defined as the difference between the weight measured at the last antenatal visit and the pre-pregnancy or booking weight (range in kg):   1. BMI underweight: 12.5-18.0 kg 2. BMI normal: 11.5-16.0 kg 3. BMI overweight: 7.0-11.5kg 4. BMI obese: 5.0-9.0kg   The calculated gestational weight gains of each pregnant mother was then compared with the recommended total pregnancy weight gain and then classified as:   1. Below IOM recommendation – inadequate weight gain 2. Follow IOM recommendation – adequate weight gain 3. Above IOM recommendation – excessive weight gain |
| Dengue infection | Present a confirmed dengue infection (positive diagnostic test such as dengue NS1 antigen test and Dengue IgM/IgG serology test) diagnosed during pregnancy identified at each time point as per the record in the antenatal booklet. It was categorised as below:   1. Yes (diagnosed at any time point) 2. No (not diagnosed at all time points) |
| COVID-19 infection | Present a confirmed COVID-19 infection (as listed in the Malaysian guideline) diagnosed during pregnancy identified at each time point as per the record in the antenatal booklet.  Case definition of a confirmed case of SARS-CoV-2 infection:   1. A person with a positive Nucleic Acid Amplification Test (NAAT), RTPCR, Rapid Molecular, and Gene X-pert 2. A person with a SARS-CoV-2 RTK-Ag AND meeting either the probable case definition or suspected criteria. 3. An asymptomatic with a positive SARS-CoV-2 RTK-Ag AND who is a contact of a probable or confirmed case.   It was categorised as below:   1. Yes (diagnosed at any time point) 2. No (not diagnosed at all time points) |
| Urinary tract infection | Present a confirmed urinary tract infection (positive bacteriuria from urine FEME analysis and/or urine culture and sensitivity test) diagnosed during pregnancy identified at each time point as per the record in the antenatal booklet. It was categorised as below:   1. Yes (diagnosed at any time point) 2. No (not diagnosed at all time points) |
| Gestational hypertension | It is characterised by a systolic pressure of ≥140 mmHg and a diastolic pressure of ≥90 mmHg without proteinuria diagnosed during pregnancy at or after 20 weeks of gestation. It was identified at each time point as per the record in the antenatal booklet and categorised as below:   1. Yes (diagnosed at any time point) 2. No (not diagnosed at all time points) |
| Preeclampsia | It refers to gestational hypertension accompanied by ≥ 1 new onset of a condition diagnosed during pregnancy at or after 20 weeks of gestation, such as proteinuria and other maternal organ dysfunction. It was identified at each time point as per the record in the antenatal booklet and categorised as below:   1. Yes (diagnosed at any time point) 2. No (not diagnosed at all time points) |
| Maternal anaemia | Defined as the blood haemoglobin concentration below 11 g/dL diagnosed during pregnancy identified at each time point as per the record in the antenatal booklet. It was categorised as below:   1. Yes (diagnosed at any time point) 2. No (not diagnosed at all time points)   (WHO, 2017) |
| Gestational diabetes mellitus | Defined according to protocol practice at the selected MCH clinics, based on the fasting plasma glucose (FPG) and 2-hour post-glucose challenge. It was identified at each time point as per the record in the antenatal booklet and categorised as below:   1. Yes (diagnosed at any time point) 2. No (not diagnosed at all time points) |
